# Supplementary material for: Synthesis and Structural Characterization of Phosphanide Gold(III)/Gold(I) Complexes and Their Thallium(III) and Gold(III) Precursors
Source: Molecules. 2023 Jan 3;28(1):447. doi: 10.3390/molecules28010447 (PMC9824866; doi:10.3390/molecules28010447)
Supplement: Supplementary file 1 [file molecules-28-00447-s001.zip › molecules-2090754-supplementary.pdf]

Synthesis and Structural Characterization of Phosphanide Gold(III)/Gold(I) Complexes and Their Thallium(III) and Gold(III) Precursors

Laura Coconubo-Guio, José M. López-de-Luzuriaga *, Sonia Moreno and M. Elena Olmos *

Departamento de Química, Centro de Investigación en Síntesis Química (CISQ), Complejo Científico Tecnológico, Universidad de La Rioja, Madre de Dios 53, 26006 Logroño, Spain

* Correspondence: josemaria.lopez@unirioja.es (J.M.L.-d.-L.), m-elena.olmos@unirioja.es (M.E.O.)

Supplementary Material

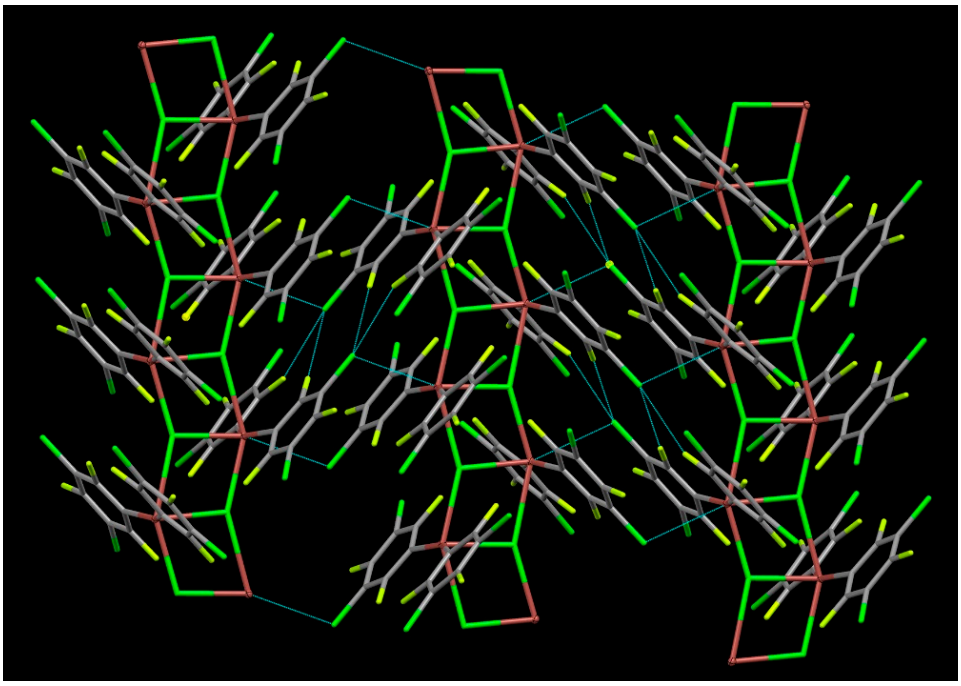

Figure S1. View of the 2D polymeric structure in [Tl(3,5-$C_6F_3Cl_2$)$_2$Cl]$_n$ (**1**) formed through Tl···Cl [3.4716(11) Å] and Cl···F contacts [3.206(3) and 2.975(4) Å] seen from the crystallographic *a* axis. Color code: C, grey; Cl, green; F, light green; Tl, brown.

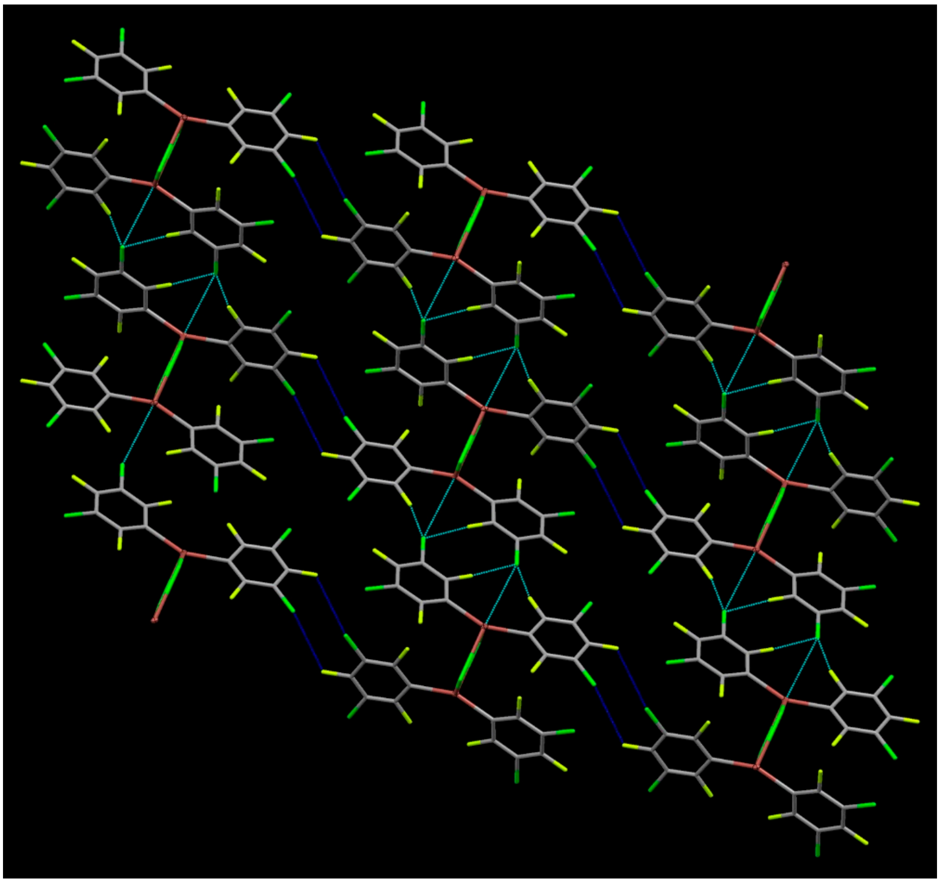

Figure S2. View of the 3D network in [Tl(3,5-$C_6F_3Cl_2$)$_2$Cl]$_n$ (**1**) formed through Cl···F contacts [3.193(4) Å] seen from the crystallographic *b* axis. Color code: C, grey; Cl, green; F, light green; Tl, brown.

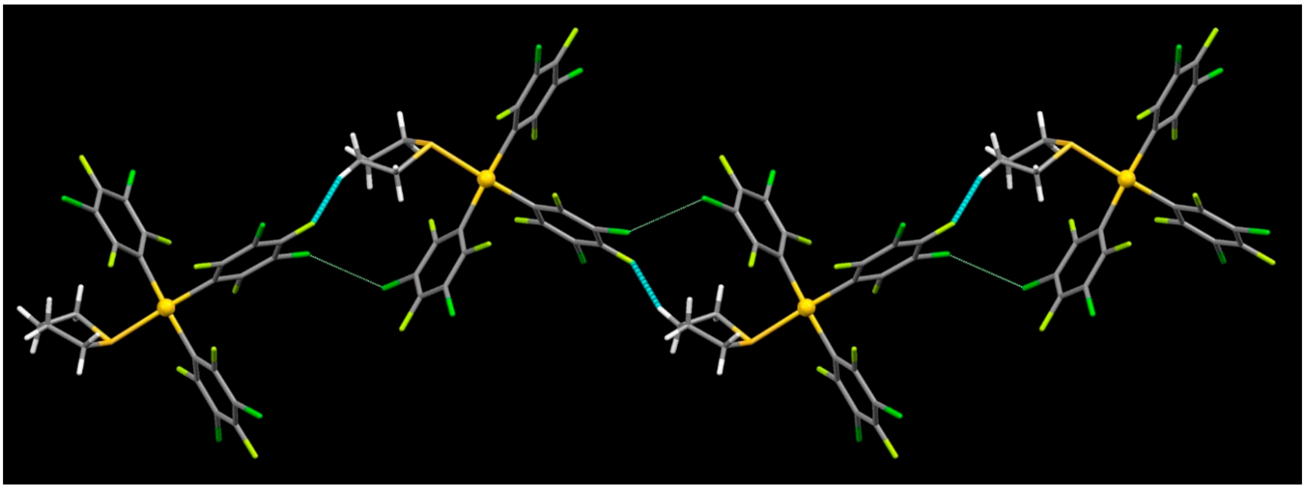

Figure S3. View of the 1D supramolecular structure in [Au(3,5-$C_6F_3Cl_2)_3$(tht)] (**2**) formed through C-H···F hydrogen bonds and Cl···Cl contacts [3.4651(12) Å] seen from the crystallographic *a* axis. Color code: C, grey; H, white; Au, yellow; Cl, green; F, light green; S, orange.

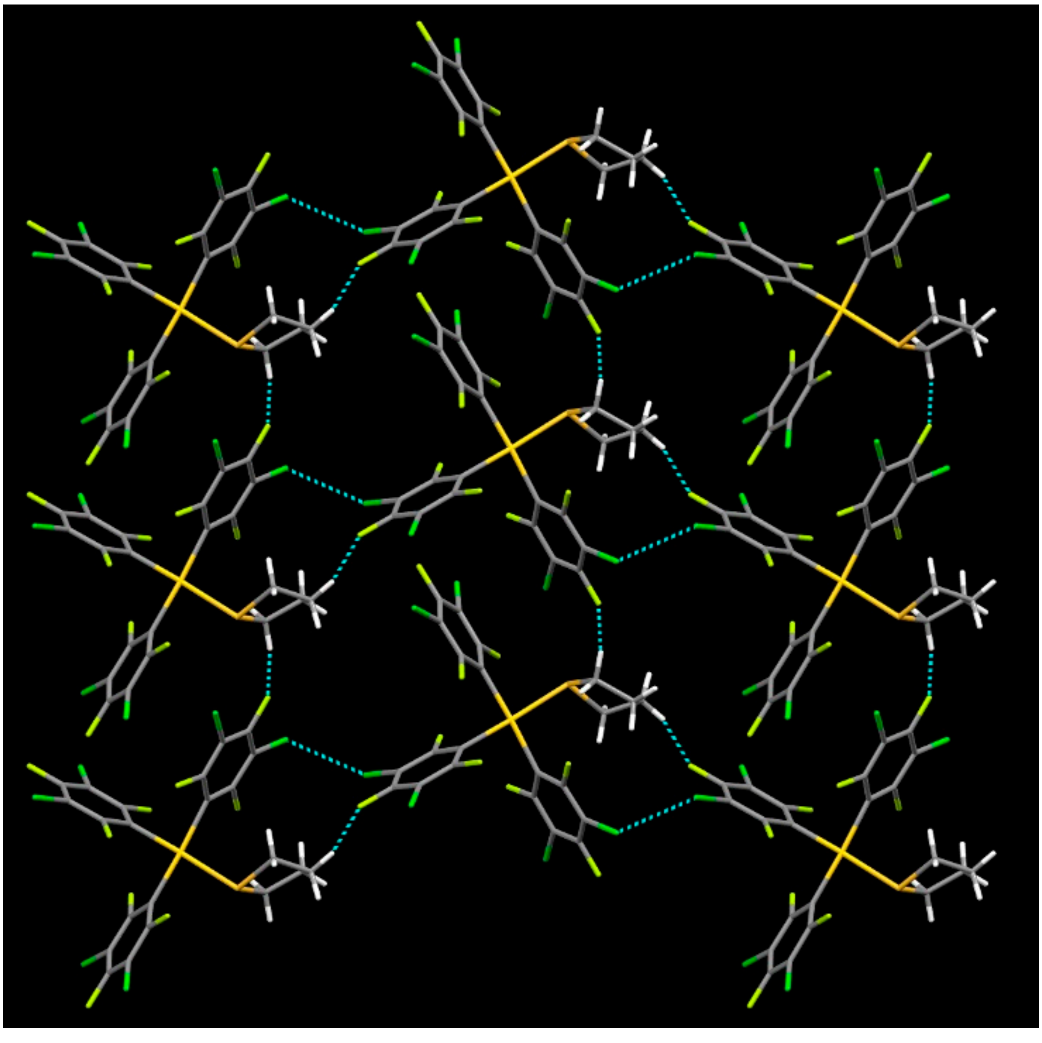

Figure S4. View of the 2D network in [Au(3,5-$C_6F_3Cl_2)_3$(tht)] (**2**) formed through C-H···F hydrogen bonds seen from the crystallographic *c* axis. Color code: C, grey; H, white; Au, yellow; Cl, green; F, light green; S, orange.

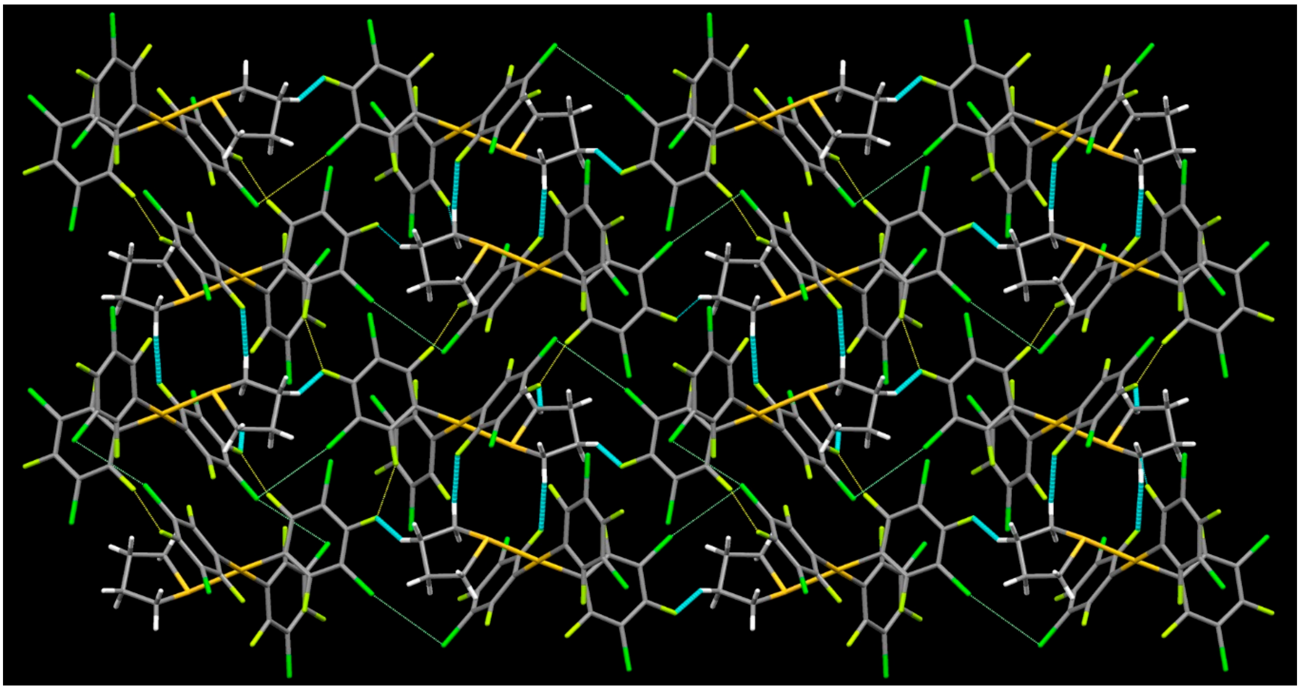

Figure S5. View of the 3D network in [Au(3,5-$C_6F_3Cl_2$)$_3$(tht)] (**2**) formed through C-H···F hydrogen bonds. Color code: C, grey; H, white; Au, yellow; Cl, green; F, light green; S, orange.

Table S1. Hydrogen bond lengths (Å) and angles (º) for [Au(3,5-$C_6F_3Cl_2$)$_3$(tht)] (**2**).

D-H···A	d(D-H)	d(H···A)	d(D···A)	<(DHA)
C(22)-H(22A)···F(8)#1	0.99	2.46	3.324(4)	145.7
C(19)-H(19B)···F(9)#2	0.99	2.37	3.123(4)	132.1
C(20)-H(20A)···F(5)#3	0.99	2.46	3.350(4)	149.5

Symmetry transformations used to generate equivalent atoms:
#1 x+1,y,z #2 -x+1,-y+1,-z+1 #3 -x+1/2,y+1/2,-z+1/2

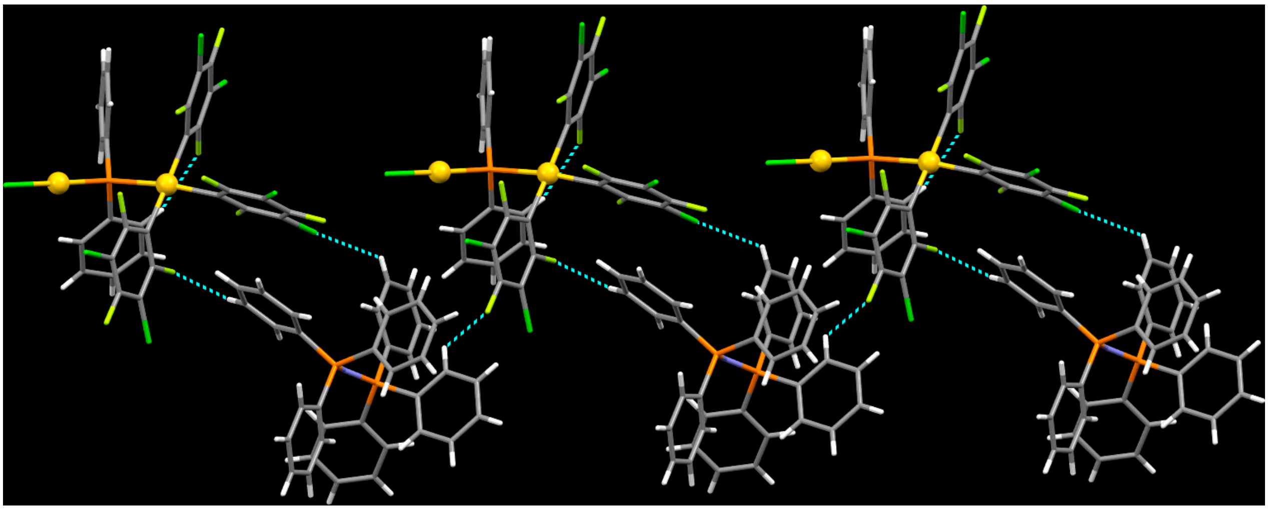

Figure S6. View of the 1D supramolecular structure in PPN[Au(3,5-$C_6Cl_2F_3$)$_3$(μ-PPh_2)AuCl] (**4**) formed through C-H···F and C-H···Cl hydrogen bonds. Color code: C, grey; H, white; Au, yellow; Cl, green; F, light green; N, violet; P, orange.

Table S2 Hydrogen bond lengths (Å) and angles (º) for PPN[Au(3,5-$C_6Cl_2F_3$)$_3$(μ-PPh_2)AuCl] (**4**).

D-H···A	d(D-H)	d(H···A)	d(D···A)	<(DHA)
C(30)-H(30)...F(1)	0.93	2.47	3.352(8)	159.1
C(42)-H(42)...F(13)#1	0.93	2.51	3.208(7)	132.4
C(35)-H(35)...Cl(7)#2	0.93	2.88	3.533(8)	128.2
C(57)-H(57)...F(11)#2	0.93	2.60	3.474(8)	156.8

Symmetry transformations used to generate equivalent atoms:
#1 x+1,y,z #2 x+1,y-1,z

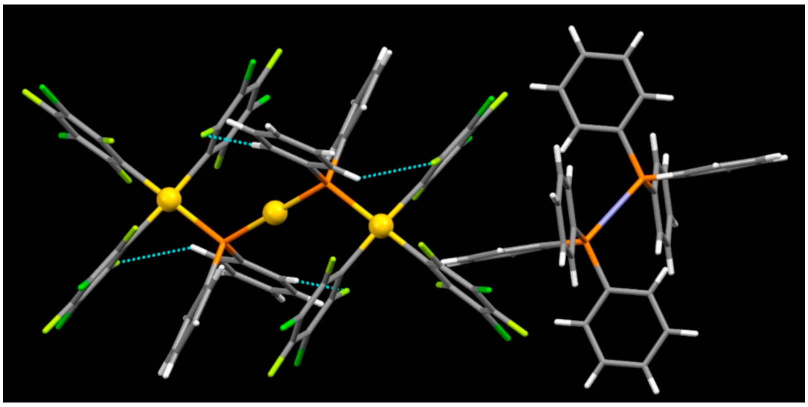

Figure S7. View of the molecular structure of the ions in PPN[{(3,5-$C_6Cl_2F_3$)$_3$Au(μ-PPh_2)}$_2$Au] (**5**) showing the intramolecular C-H···F hydrogen bonds. Color code: C, grey; H, white; Au, yellow; Cl, green; F, light green; N, violet; P, orange.

Table S3. Hydrogen bond lengths (Å) and angles (º) for PPN[{(3,5-$C_6Cl_2F_3$)$_3$Au(μ-PPh_2)}$_2$Au] (**5**).

D-H···A	d(D-H)	d(H···A)	d(D···A)	<(DHA)
C(20)-H(20)···F(9)#1	0.95	2.52	3.462(6)	171.1
C(24)-H(24)···F(1)	0.95	2.62	3.427(6)	143.7

Symmetry transformations used to generate equivalent atoms:
#1 -x+1,-y+1,-z+1 #2 -x+1,-y,-z+2
